# Anomalous Magnetic Orientations of Magnetosome Chains in a Magnetotactic Bacterium: *Magnetovibrio blakemorei* Strain MV-1

**DOI:** 10.1371/journal.pone.0053368

**Published:** 2013-01-08

**Authors:** Samanbir S. Kalirai, Dennis A. Bazylinski, Adam P. Hitchcock

**Affiliations:** 1 Department of Chemistry and Chemical Biology, McMaster University, Hamilton, Ontario, Canada; 2 School of Life Sciences, University of Nevada at Las Vegas, Las Vegas, Nevada, United States of America; University of Waterloo, Canada

## Abstract

There is a good deal of published evidence that indicates that all magnetosomes within a single cell of a magnetotactic bacterium are magnetically oriented in the same direction so that they form a single magnetic dipole believed to assist navigation of the cell to optimal environments for their growth and survival. Some cells of the cultured magnetotactic bacterium *Magnetovibrio blakemorei* strain MV-1 are known to have relatively wide gaps between groups of magnetosomes that do not seem to interfere with the larger, overall linear arrangement of the magnetosomes along the long axis of the cell. We determined the magnetic orientation of the magnetosomes in individual cells of this bacterium using Fe 2p X-ray magnetic circular dichroism (XMCD) spectra measured with scanning transmission X-ray microscopy (STXM). We observed a significant number of cases in which there are sub-chains in a single cell, with spatial gaps between them, in which one or more sub-chains are magnetically polarized opposite to other sub-chains in the same cell. These occur with an estimated frequency of 4.0±0.2%, based on a sample size of 150 cells. We propose possible explanations for these anomalous cases which shed insight into the mechanisms of chain formation and magnetic alignment.

## Introduction

Magnetotactic bacteria (MTB) are ubiquitous in marine and freshwater environments [1], [2], [3]. They are a diverse group phylogenetically and morphologically which are linked by the ability to biomineralize membrane-bounded magnetic nanoparticles termed magnetosomes. Magnetosomes are single-domain magnetic crystals of either magnetite, Fe_3_O_4_, or greigite, Fe_3_S_4_ [4], typically oriented in one or more chains. The magnetosomes are responsible for a behaviour called magnetotaxis in which cells passively align and swim along the Earth’s geomagnetic field lines, which are inclined, except at the equator [5]. By reducing a three-dimensional search problem to one of a single dimension, magnetotaxis allows for motile bacteria to more efficiently locate and maintain position at an optimal chemical environment, generally the oxic-anoxic interface, in aquatic habitats characterized by vertical chemical (e.g., oxygen) concentration gradients [5]. Thus in chemically-stratified habitats, MTB appear to have a significant advantage over non-magnetotactic bacteria in locating their preferred environment [6].

Magnetite-producing MTB synthesize magnetised chains of generally closely spaced, coherently aligned magnetosomes in order to maximize the dipole interaction to the Earth’s magnetic field [7], [8], [9]. The ability to reproducibly manufacture magnetosome magnetite crystals with high chemical purity, tight size-distribution and uniform shape represents an exquisite process of biomineralization [10], [11]. The size distribution of magnetosome crystals is highly controlled to be within the single-domain size regime [11] thereby maximizing the individual dipole moment of each magnetosome and preventing adverse size-dependent effects such as superparamagnetism and multiple domain formation which eliminate or lessen the efficacy of the magnetic particle.

There is great interest in understanding biomineralization and its associated processes, both from a fundamental perspective and also for biomimetic applications [12], [13]. Thus, shortly after the initial discovery [14], [15] of magnetotactic bacteria, an enormous effort to understand the phenomenon in depth began and it is still underway today. Much of the effort to understand magnetosome biomineralization has involved genetic techniques which has not only resulted in the discovery of genes involved in the structure, formation and organization of magnetosomes (the *mam*, *mms* and *mtx* genes) but also in finding that most of these genes are located as clusters within the genome that are further organized as a magnetosome gene island [16]. Techniques such as transmission electron microscopy (TEM) have been employed to fill the knowledge gap that is related to the understanding of the role of individual *mam* genes as well as a generic understanding of chain growth and interactions among individual magnetosomes within a chain [17].

The final magnetosome chain organization is dependent on a number of complex processes including the biological control of the growth and assembly of magnetosomes. Furthermore magnetic interactions of particles play an important role in the final observed organization of magnetosome chains. TEM [18] and electron holography measurements [18], [19] have shown that the magnetic interactions that dictate the magnetic anisotropy present in magnetosome chains are largely due to dipolar interactions of magnetosomes whereas alignment along the magnetic easy axis plays a significantly smaller role. Electron holography was also used to map the magnetic microstructure of a MV-1 magnetosome chain [7]. That study, the first to map the magnetic moment of a single magnetosome chain, measured a moment of 7×10^−16^ Am^2^ for a chain containing 15 magnetosomes (∼1600 nm long) [7]. Several studies have shown that some magnetosome proteins play an important role in magnetosome chain organization. *Mam*J has been shown to anchor magnetosomes to a filament while *Mam*K [20], [21] is required for magnetosome organization into a single chain. Scheffel *et al.* [20] showed through cryo-electron tomography measurements that Δ*Mam*J deletion mutants of *Magnetospirillum gryphiswaldense* show no organization of magnetosomes despite the presence of cytoskeletal filaments which magnetosomes anchor onto in wild type cells. Komeili *et al.* [21] used cryo-electron tomography to show that the cytoskeletal filament is composed of *Mam*K by comparing wild-type cells of *Magnetospirillum magneticum* to the Δ*Mam*K deletion mutant, which showed no chain like organization of magnetosomes. Furthermore Komeili *et al.* showed that magnetosomes are invaginations of the cell membrane [21], possibly to overcome magnetic interactions of free magnetosomes which would preferentially organize into aggregates. Klumpp and Faivre [22] simulated the dynamics of magnetosome chain formation, accounting for biological regulation and magnetic interaction factors using a stochastic model. This model suggests that the organization of magnetosomes into chains is best explained when both magnetic interactions and biologically induced active movement within the cell are taken into account [22]. This coordination allows for organization of magnetosomes into a single chain with a unidirectional polarization. Interestingly, when magnetosome organization is dictated by the magnetic interactions and biological control is removed, the simulation yields results where magnetosome sub-chains appear with opposing polarities. The authors propose this is an analogous model to Δ*Mam*K deletion mutants [22]. Thus magnetic interactions alone have been shown to be insufficient in creating single chain organization. Rather, scaffolding and active movement related to biological control are necessary [22].

Here we report a study that uses Scanning Transmission X-ray Microscopy (STXM) [23]–[25] and X-ray Magnetic Circular Dichroism (XMCD) [26] to determine the magnetic and chemical properties of magnetosome chains in individual cells of the magnetotactic bacterium *Magnetovibrio blakemorei* strain MV-1 [27] at the individual magnetosome level [28], [29]. Whereas most prior studies have focused on chains with regularly spaced magnetosomes, in which the magnetic vector of each magnetosome is aligned in the same direction as that of all other magnetosomes in the chain, we have focused on cells which contain sub-chains separated by spatial gaps. MV-1 was chosen as a model organism because of prior observations of spatial gaps between magnetosome sub-chains in these cells [7], [30]. Our STXM-XMCD results show that, unlike the majority of magnetosome chains studied to date, sub-chains separated by gaps in MV-1 can have opposing magnetic orientations. Spatial gaps are defined as gaps that are larger than 50 nm which is much larger than typical magnetosome separation distances in condensed chains. Our study of these cases provides additional insight into the mechanisms of chain formation.

## Experimental

### STXM-XMCD Measurements

STXM-XMCD measurements were made at the Canadian Light Source (CLS) 10ID-1 spectromicroscopy (SM) [31] and the Advanced Light Source (ALS, Lawrence-Berkeley National Lab – LBNL) 11.0.2 [32] beamlines. These two beamlines have similar optical designs. The light source for each line is an elliptically polarizing undulator (EPU) which can switch the polarization of the light radiated in the Fe 2p (L_23_) region (700–740 eV) from pure circular left to pure circular right (at the CLS), or from 90% circular left to 90% circular right (at the ALS). In each beamline an infinity focused plane grating monochromator produces monochromated X-rays with high resolving power (>10,000). The energy scale was calibrated by measuring the F 1 s spectrum of SF_6_ [33] at a time displacement relative to the MV-1 measurements that was within the time period that energy scales are stable on the CLS beamline. A Fresnel zone plate (outer zone width of 25 nm, diameter of 240 µm, 95 µm diameter central stop, provided by the Centre for X-ray Optics, LBNL) focuses the monochromated light to a nominal spot size of 30 nm. The zero order and higher order diffracted light are blocked by an order sorting aperture. The sample is mounted with a rotation of 60° in the horizontal direction relative to the x-ray propagation axis, in order to have a projection of the in-plane magnetic vector of the sample on the photon spin axis. The region of interest is placed at the focal point of the first-order diffracted light and the sample is raster scanned using the interferometrically controlled sample stage. The transmitted X-rays are converted to visible light by a scintillator and counted with a high performance photomultiplier tube (maximum count rate of 20 MHz). The signal is processed to form an image on a pixel-by-pixel basis. Once a complete image is formed, the polarization is switched to image the same region, at the same energy, with oppositely polarized light. Once images with both polarizations have been recorded the photon energy is stepped [29]. At the end of the XMCD measurement two stacks, one for each polarization are created. A stack is a three-dimensional data construct with the transmitted intensity at each pixel (x, y) at each energy (E) and for a specific polarization. After image alignment, the total transmitted intensity profile, **I**(x,y,E), is converted to an optical density (OD = ln(I/I_0_)) stack, using the Lambert-Beer Law and incident flux (I_0_), which is measured at a point adjacent to the MTB cell under study. Optical density stacks are an array of NEXAFS spectra which can be analyzed to give spatially-resolved speciation information. The use of polarization-dependent stacks gives the additional ability to discern chemically specific magnetic information via X-ray magnetic circular dichroism (XMCD) spectroscopy [26] where the XMCD signal is defined as
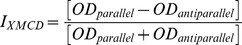
(1)


Additionally one can obtain magnetically sensitive XMCD maps, which are the difference of images recorded with each polarization at a single, magnetically sensitive photon energy. A photon energy of 708.2 eV, the energy of the first minimum in the XMCD spectrum, provides maximum magnetic sensitivity in the case of magnetite.

### Preparation of Magnetotactic Bacteria

Cells of *Magnetovibrio blakemorei* strain MV-1, the magnetotactic bacterium under study, were grown anaerobically in liquid cultures with nitrous oxide as the terminal electron acceptor as previously described [34]. Cells were harvested from cultures at mid- to late-exponential phase of growth.

### Transmission Electron Microscopy

Cells were deposited on standard formvar coated copper electron microscope grids, washed to remove culture salts, and viewed with a JEOL Model JEM 1200 EX transmission electron microscope.

## Results

**Figure 1:**
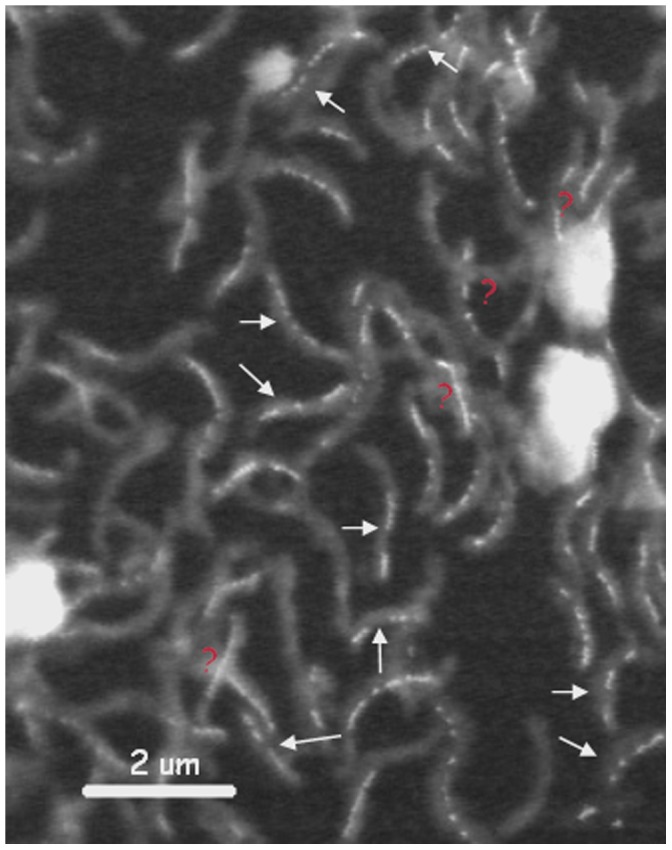
STXM image of an ensemble of cells of *Magnetovibrio blakemorei* strain MV-1 deposited on a formvar coated 3 mm transmission electron microscopy (TEM) Cu grid. The image was recorded at 709.8 eV and converted to optical density (OD) using the intensity in areas without cells in this image. The arrows indicate cells with apparent gaps in their magnetosome chains. The question marks (red) indicate (multiple) cells that were discounted because of uncertainty as to the number of cells and/or magnetosome chains present.

**Figure 1** shows a STXM image of a number of cells of *Magnetovibrio blakemorei* from a cultured sample. The image was recorded in transmission using a photon energy of 709.8 eV and converted to optical density (OD) using the intensity in areas without cells in this image. In addition to the cells with clearly visible chains of magnetosomes, this field of view contains a number of unstructured features of amorphous material, of unknown character but likely either extracellular material from the MV-1 cells or residual material from the culturing medium. In the field of view in Fig. 1 there are a significant number of MV-1 cells that do not contain magnetosomes and others that contain chains that are interrupted by gaps and/or have poorly formed chains. The frequency of cells without magnetosomes and those with interrupted chains was measured from this and other, similar images. In a sampling of 351 cells, 29% had no magnetosomes while 39% of the cells had gaps in their magnetosome chains. The number of cells without magnetosomes is high compared to observations on other cultures and species of magnetotactic bacteria but this should not be looked at as indicating an unhealthy MV-1 culture. Cells reduced nitrous oxide as the terminal electron acceptor under chemo-organo-heterotrophic conditions and reached normal cell yields at the end of growth, supporting the fact that the cultures behaved normally. Moreover most cells still biomineralized magnetosomes. Finally, although the fraction without magnetosomes is rather large, it is similar to that of other MV-1 cultures we have examined over the past 4 years. Although quantitative information is not available for all those cultures, we feel the sample set we used to perform the statistical analysis reasonably exemplifies the family of cultures we have been studying.

From a 150 cell subset of these 351 cells for which the XMCD was measured, there were 6 cells containing two or more sub-chains of magnetosomes with opposite magnetic polarities, resulting in a frequency of magnetic anomalies of 4.0±0.2% of the total cell population, and a 9.9±0.4% frequency with respect to those cells with magnetosome chain gaps. Magnetic anomalies are defined as magnetosome chains with at least one magnetosome-sized gap in the chain (>50 nm), with the gap separating sub-chains with opposite magnetic orientation.

**Figure 2:**
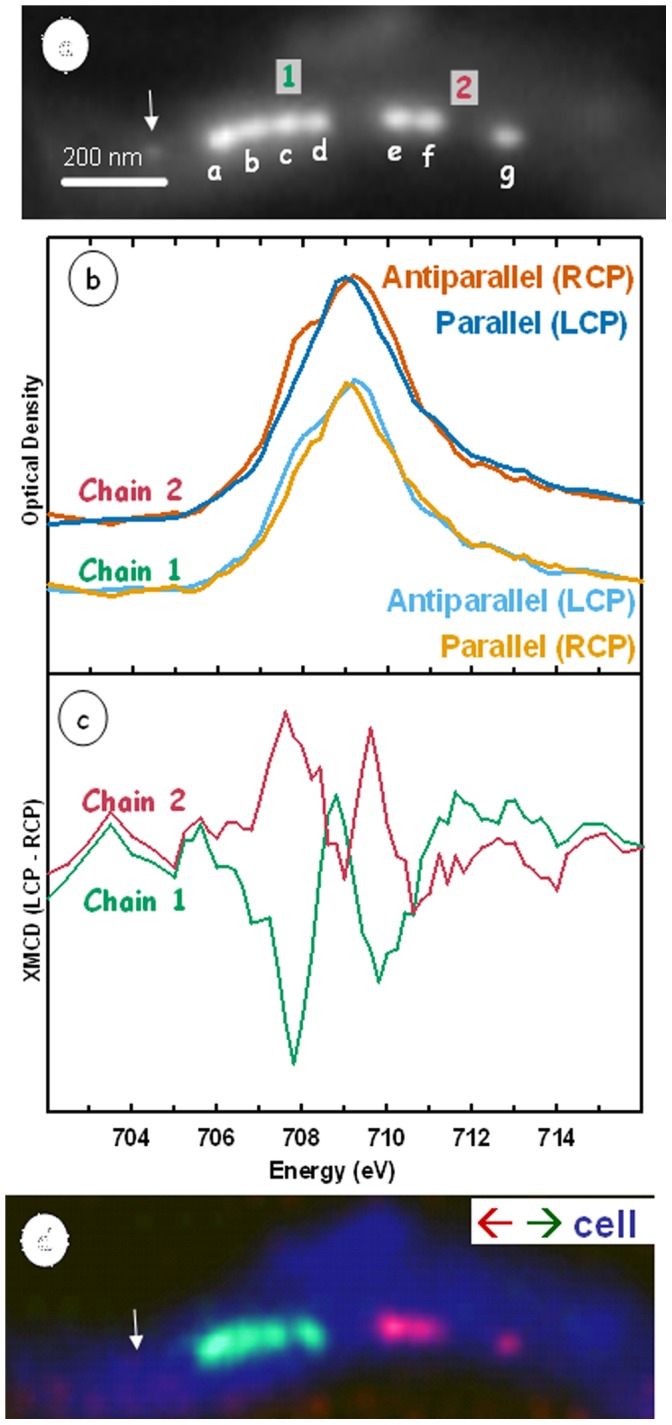
X-ray magnetic circular dichroism (XMCD) of an MV-1 cell with a chain of magnetosomes containing two gaps. (a) STXM image (OD). The two sub-chains are labelled chain 1 and chain 2. Each magnetosome has been labelled a-g. (b) Fe 2p_3/2_ spectra from the left chain (upper) and the right chain (lower) recorded with the two circular polarizations. (c) XMCD signals derived by subtracting the two circular polarization spectra. (d) Color coded composite of the cell (OD image at 704 eV) (blue), magnetosomes with a leftward polarization (green), and a rightward polarization (red). The arrows in Figs (a) and (d) indicate a ‘pre-magnetosome’ – see text.

**Figure 2a** shows a single MV-1 cell containing a characteristic interrupted chain of magnetosomes. Figure 2b and 2c present the Fe 2p_3/2_ X-ray absorption spectra for the two chain sections, and their associated XMCD signals respectively. The spectral signals show there is an inversion of the magnetic signal of one with respect to the other. Fig. 2d presents a color coded composite of the XMCD signals with that for the cellular material, as visualized at 704 eV, below the onset of the Fe 2p edge. This presentation clearly shows that the magnetic orientation of the chain section on the left is opposite to that of the chain section on the right. The superposition of two cells with separate chains is ruled out due to the pre-edge average image (blue in Fig. 2d) which is sensitive to the cell density. If two cells were superimposed in this image the pre-edge signal at the intersection would be discernibly larger than at the ends of the chain where the image is clearly that of an individual cell.

**Figure 3:**
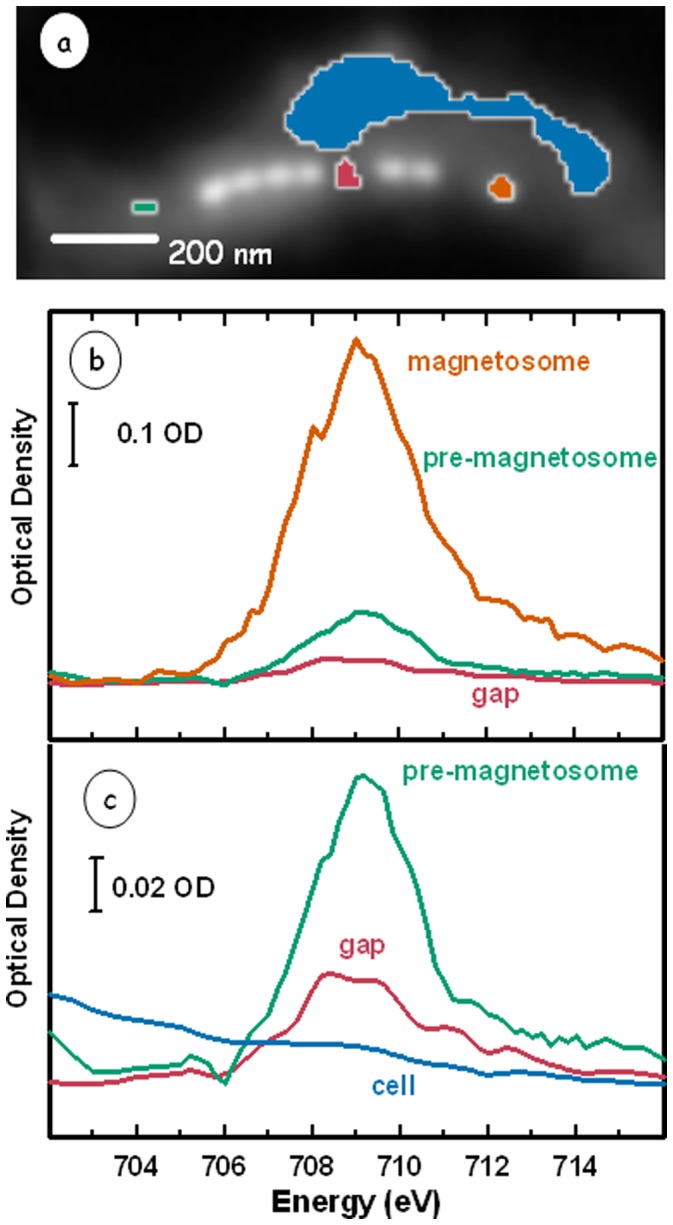
STXM image at 709.8 eV of an MV-1 cell with two gaps along the magnetosome chain. (b) Fe 2p_3/2_ spectra (average of left and right circular polarization data) of different regions of the cell: a single magnetosome (red), the first gap (pink), the “pre-magnetosome” (green), and the cytoplasm of the cell (blue) away from the magnetosome chain. (c) Expansion of the gap and pre-magnetosome spectra. The vertical lines indicate energies traditionally associated with Fe(II) and Fe(III) oxidation state signals.

The Fe 2p_3/2_ spectrum of the gap between two magnetosome sub-chains (red); that of an immature, possibly super-paramagnetic, magnetosome crystal (green); that of the cell cytoplasm (blue), and that of a single magnetosome (orange), are compared in **Figure 3**. This comparison shows that there is more iron within the gap than in the parts of the cell outside the magnetosome chain. Furthermore the spectral shape of the iron within the gap is distinct from that of the mature and immature magnetosome magnetite crystals. Signals at ∼708 eV and ∼710 eV are traditionally associated with Fe(II) and Fe(III) oxidation states respectively (although the spectra of pure Fe(II) and pure Fe(III) species contain some signal at each energy). The gap area shows a higher Fe(II) content than that of the mature magnetosome crystal. The Fe 2p_3/2_ spectrum of the mature magnetosome crystals matches well with that of reference magnetite [29]. The Fe 2p_3/2_ spectrum of the immature magnetosome crystal is similar to that of a mature one but it is non-magnetic (zero XMCD).

**Figure 4:**
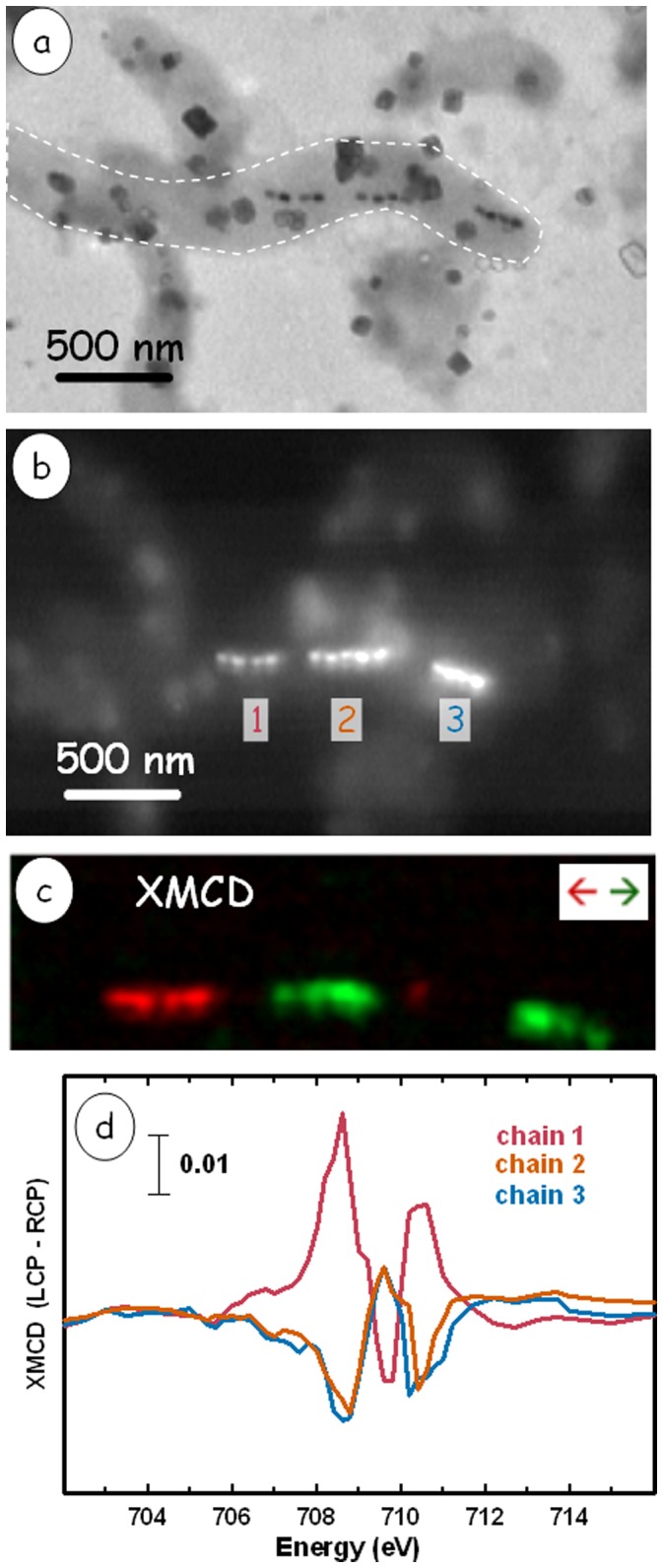
XMCD of an MV-1 cell showing gaps in the magnetosome chain. (a) TEM image of the cell. The rectangular dark objects are salt crystals from the growth medium. The dashed white line indicates the boundary of the cell which contains 3 sub-chains. (b) STXM image at 709.8 eV of the identical region. (c) Color coded composite of the XMCD signal from the magnetosome chain. (d) XMCD spectra of the 3 subchains.

**Figure 4**a shows a TEM image of a whole intact MV-1 cell while Figure 4b is the corresponding STXM image of the same cell at 709.8 eV. There are three magnetosome sub-chains in this cell, with gaps between them. The XMCD map of the three sub-chains (Figure 4c) shows that there is an opposite magnetic orientation between chain 1 and chains 2 & 3. This is reflected in the opposite sense of the XMCD spectra for the three sub-chains (Figure 4d). On average, the XMCD signal of chain 1 is larger than that of chains 2 & 3.

## Discussion

Figure 3c shows that the distribution of iron species in the gap has more ferrous, Fe(II) content than magnetite. Previous studies have shown that the form of iron that is transported to magnetosome vesicles to form biogenic magnetite is ferrous in nature [35], [36], [37]. Any ferric iron transport would most likely involve siderophores to prevent any Fe(III) from precipitation [38]. Frankel et al. [37] used ^57^Fe Mössbauer spectroscopy to examine the nature and distribution of major iron compounds in *M. magnetotacticum*. They proposed a model in which Fe(III) is taken up by the cell by non-specific means, reduced to Fe(II) as it enters the cell, then re-oxidized to Fe(III) oxide during formation of magnetosomes.The fact that the Fe 2p_3/2_ spectral signature found in the gap has a more ferrous character than the magnetosomes, is consistent with this notion. That iron signal may be from a magnetosome vesicle that has either failed to precipitate magnetite, or is just prior to magnetite formation.

The finding of magnetotactic bacterial cells containing small chains of magnetosomes of opposite polarity has not been reported previously to our knowledge. It is an uncharacteristic observation relative to the present understanding of the function of the magnetosome chain in magnetotactic bacteria [2]. In particular, if the number of magnetosomes in each section are approximately the same, the opposite magnetic orientation of the two chain sections would lead to a cancellation of their magnetic moments. Thus the net magnetic moment of the cell would be much smaller, possibly to the point where the interaction with the earth’s magnetic field would be insufficient to impose a spatial orientation. The net field expected for the magnetic arrangement in the cell in fig. 2 is approximately equivalent to that of only one magnetosome [39], which would results in an interaction with the earth’s field that is only 20% of that if all the magnetosomes had the same magnetic orientation. Thus, the cell in fig. 2 would experience negligible spatial alignment with the earth’s field. While this may be detrimental to the individual cell, since there are a large fraction of cells without any magnetosomes at all, it seems that this situation does not lead to cell death or to inhibit growth. What is interesting to consider is the significance of our observations with respect to the mechanisms of magnetosome chain formation.

One possibility that might explain the observation is that the anomalous cells experienced a high external magnetic field that has reversed the magnetic orientation of one half of a partial chain, without affecting the other half. This is very implausible since a large, in-plane magnetic field (∼6.37×10^4^ A/m) is required to reverse the moment of a single domain magnetite crystal [9]. If the sample was exposed to external fields of this magnitude one would expect both sub-chains to be influenced similarly. A second possibility is that the magnetosome chain was influenced by the field of an oppositely oriented chain in a cell which came into close proximity. However, the field generated by a magnetosome chain external to the cell is too weak to modify the magnetization of magnetosomes inside an adjacent cell because of the large field of the intracellular chains and the rapid drop of the strength of the magnetic field with increasing distance. Previously, coercive field strengths of 1.90×10^3^ to 2.85×10^3^ A/m were found to be necessary to switch the polarity of a MV-1 chain of 15 magnetosomes [7]. However, this may vary as the coercive fields of magnetosome chains are a function of the chain length, the inter-particle spacing, and the size of the magnetosomes [40].

Distances were calculated from the images shown in figures 2a and 4b. Distances of the gaps from left to right in figure 2a are 107±8 nm and 96±8 nm (the foreshortening due to the tilt of the sample has been taken into account). For figure 4b, the distances from left to right are 150±7 nm, 46±13 nm and 242±17 nm. Calculations based on a simple point magnetization model were performed using the chain setup seen in figure 2a. The magnetic moment of a chain of 15 magnetosomes in an MV-1 cell was calculated to be 7.1×10^−16^ Am^2^ from electron holography measurements [7]. Based on this result, the average magnetosome magnetic moment is estimated to be 4.5×10^−17^ Am^2^ for each of the 4 magnetosomes in Fig. 2. The field at “magnetosome e” in chain 2 from the adjacent 4-magnetosome chain is approximately 5.5×10^4^ A/m in the direction of its experimentally determined moment. Without the presence of “magnetosome f” the field at the magnetosome would be ∼4.4×10^3^ A/m in the opposite direction of the moment. In the absence of a correspondingly oriented magnetosome, this field should be strong enough to reorient “magnetosome e” according to prior studies on the magnetic hysteresis of magnetosomes [7].

A number of interpretational comments are given. Firstly, these observations indicate that magnetosome particles may have well spaced nucleation sites or be inherited during cell splitting when cells are elongated and field strengths are not high enough to cause preferential alignment of magnetic moments. Secondly, for the magnetosome sub-chains not to be influenced by neighbouring sub-chains and re-orient, the sub-chain must consist of more than one magnetosome to maintain the opposing orientation by creating a large enough local magnetic field. Thirdly, the observed separation distance in the cases presented here signifies that there is active transport of magnetosomes using non-magnetostatic interactions. Previous studies have shown that proteins allow for and are necessary for correct magnetosome chain formation via a non-magnetostatic magnetosome formation [20], [21], [22]. It has been postulated that the filaments required to congregate magnetosomes into a single chain produce forces which may additionally be aided by magnetostatic interactions [41], [42], [43]. If magnetosomes adopt an opposite orientation then the magnetostatic force between them becomes repulsive. At a separation distance of ∼50 nm the repulsive force is approximately 50 pN [44]. This force increases as the separation distance is decreased, thus at some distance, filament derived forces driving magnetosomes into a single chain equilibrate with the repulsive forces between oppositely oriented sub-chains necessitating a gap between oppositely oriented magnetosomes.

During cell replication, if the cell divides part way through a chain of magnetosomes, newly formed magnetosome crystals should continue to have the same magnetic orientation if biomineralized occurs at the ends of the partial magnetosome chain that the cell inherited (providing that there are no gaps between magnetosomes). One reasonable explanation of the opposing magnetic moment of the sub-chains could then be that they are a consequence of newly formed magnetosomes which are produced within the cell. If the newly forming magnetosomes were produced at a distance where the field from the original magnetosome chain inherited from cell division was insufficient to align the magnetic vector of the new magnetosomes, their magnetic orientation would be determined by other factors, such as the earth’s field or other local magnetic fields. Winklhofer et al, [45] have calculated that the degree of preferential alignment with the earth’s field at a field strength of 0.25 G is 0.54∶0.46, while at a field of 0.50 G the preferential alignment is 0.58∶0.42. Of course fields from adjacent magnetosomes will overcome the alignment forces from the earth’s field. At a distance of 0.25 µm (5 times the diameter of a magnetosome) the field strength is such that the preferential alignment is 0.9∶0.1– i.e. only 10% of magnetosomes formed at such a distance would have an anti-parallel magnetization.

A more likely scenario is that gaps between magnetosomes or magnetosome chains are due to multiple nucleation sites for magnetosomes in the cell. If the gap is large enough, a pre-existing chain would have little effect on magnetosomes forming at other nucleation sites provided there is an appropriate separation distance. The newly forming magnetosome crystal would have a near 50∶50 chance of being of either polarity (see preceding paragraph). Those that form adjacent, or within the range where the magnetostatic interaction is large enough to coerce newly formed magnetosomes, form a chain with the same polarity. In *Magnetospirillum gryphiswaldense*, it has been shown that magnetite is nucleated at discrete sites that are scattered throughout the cell and only undergo a localization at a later stage of magnetosome growth [20], [46]. Conversely, in *Magnetospirillum magneticum* it has been observed that magnetosome membranes are localized even prior to magnetosome formation [21]. Thus there is evidence for both a localized and delocalized nucleation model for magnetosome chain growth. It may be that the magnetic field from one sub-chain at another magnetosome that is separated from the chain by a gap is too small for one chain to affect the magnetic orientation on the opposite chain. It is difficult to tell from the XMCD maps, as compared to higher resolution XMCD stacks, which were used to determine occurrences of sub-chains with opposing moments, whether the distances are sufficient to allow for magnetosome chains with opposite magnetic orientations.

Komeili *et al.* [21] and Scheffel *et al.* [20] showed that, in cells of *Magnetospirillum* species, magnetosomes are localized and assembled by the filamentous actin-like protein MamK that appears to make up a prokaryotic cytoskeleton responsible for the stability of the magnetosome chain. An acidic magnetosome membrane protein apparently is responsible for anchoring the magnetosome to MamK [20]. In addition, it is thought that magnetosomes are aggregated in part by magnetic interactions [20]. Cells of *Magnetovibrio blakemorei* contain a *mamK* gene in their genome but not *mamJ*. This might suggest that the mechanism for magnetosome chain formation is slightly different in *Magnetovibrio blakemorei* from that in *Magnetospirillum* species and may be the reason for the presence of gaps between magnetosomes in MV-1.

The frequency of cells with magnetosome sub-chains with opposite magnetic orientation is low. Our statistical analysis indicates 4.0% of all cells, and 9.9% of those cells which contain a gap. However, because the XMCD experiment yields magnetic information only for horizontally oriented chains (the XMCD intensity drops by the cosine of the deviation from horizontal) our frequency is most likely an underestimation. We estimate that the XMCD signal can only be observed if the chain is horizontal within ±20° due to this attenuation and the statistical limits of our measurements. Taking into account this factor, the estimated frequency of this phenomenon for MV-1 would increase from 4.0±0.2% to 18.0±0.9% when considering the whole population of MV-1 and from 9.9±0.4% to 44.6±1.8% for MV-1 cells with magnetosome chains containing gaps. Lastly, it is not known whether MV-1 cells in nature have gaps between magnetosomes and/or magnetosome chains and thus whether they have chains with opposite magnetic orientations. This is important because at this time, we cannot exclude the possibility that the gaps are an artefact due to growth conditions and/or growth rate. More growth experiments, along with observations on uncultivated, wild samples, are needed to investigate this possibility.

### Conclusions

We have shown that some cells of *Magnetovibrio blakemorei* which contain magnetosome sub-chains separated by spatial gaps show anomalous magnetic orientations. The frequency of this has been shown to be at least 4.0% of all cells and 9.9% of those cells that have gaps. The actual frequency is likely considerably higher given the nature of our measurements. All observed orientation reversal cases are associated with a gap, suggesting that the anomalous orientation of the sub-chains is due to insufficient magnetostatic interactions between the sub-chains separated by gaps.
